# Socioemotional factors associated with teacher resilience in Colombian communities affected by armed conflict a cross-sectional study

**DOI:** 10.1007/s44192-026-00449-w

**Published:** 2026-05-22

**Authors:** Lina María González-Ballesteros, Camila Andrea Castellanos-Roncancio, Oscar Eduardo Gómez Cardenas, Jennifer Clavijo-Marín, Luis Eduardo Mojica Ospina, Luis Alberto López-Romero

**Affiliations:** 1 Program in Clinical Epidemiology, Department of Clinical Epidemiology and Biostatistics, Faculty of Medicine, Pontificia Universidad Javeriana, Carrera 7 N. 40 - 62, Bogotá, Colombia; 2 Department of Psychiatry and Mental Health, Pontificia Universidad Javeriana, Bogotá, Colombia; 3 Mental Health and Resilience Research Seedbed, Department of Psychiatry and Mental Health, Faculty of Medicine, Pontificia Universidad Javeriana, Bogotá, Colombia; 4 Fundación Saldarriaga Concha, Bogotá, Colombia; 5 Faculty of Psychology, Pontificia Universidad Javeriana, Bogotá, Colombia

**Keywords:** Mental health, Teacher’s resilience, Sociodemographic factors, Psychological well-being

## Abstract

**Introduction:**

Mental health problems are increasingly prevalent among school-aged children and adolescents, underscoring the need for teacher-inclusive mental health interventions.

**Objectives:**

To examine the association between sociodemographic and socioemotional factors and resilience among teachers, prior to their participation in a school-based intervention aimed at strengthening socioemotional competencies.

**Methods:**

A cross-sectional analytical study was conducted among 693 teachers from 56 public schools in Amazonas, Boyacá, and Vaupés, Colombia. Teachers completed standardized instruments including the Connor-Davidson Resilience Scale (CD-RISC), Compassion Scale (ECOM), and Prosocial Personality Battery (PSB), along with mental health screeners for anxiety (HARS), depression (Whooley), and PTSD (PCL-C). Descriptive statistics, bivariate analyses, and multivariate linear regressions were used to assess associations between socioemotional factors and resilience scores.

**Results:**

The mean age was 47.13 years (SD = 9.91); most were female (65.95%). Median resilience score was 76 (IQR = 69–86). Teachers from Vaupés showed higher resilience, while those from Boyacá had lower scores. Higher compassion (ECOM: β = 0.23; 95% CI: 0.10–0.36) and prosociality scores (PSB: β = 0.43; 95% CI: 0.31–0.54) were independently associated with increased resilience. Conversely, higher anxiety levels (HARS: β = −0.22; 95% CI: −0.39 to − 0.06) and a positive depression screen (Whooley: β = −2.46; 95% CI: −5.18 to 0.25) were associated with lower resilience scores. Age and sex were not independently associated with resilience in the adjusted model.

**Conclusions:**

Mental health programs in school settings should prioritize teacher well-being as a central component for promoting student mental health outcomes. Findings underscore the relevance of addressing socioemotional skills and resilience in educators, particularly in contexts affected by armed conflict.

**Supplementary Information:**

The online version contains supplementary material available at 10.1007/s44192-026-00449-w.

## Introduction

The increase in the prevalence of mental disorders in children and adolescents is a problem of marked regional and global relevance [1, 2]. In Colombia, the 12-month prevalence of at least one mental disorder among children aged 7–11 years is 4.7%, with higher rates observed among those from households living in poverty [3]. In low- and middle-income countries such as Colombia the situation is further compounded by a critical shortage of specialized professionals, which limits the health system’s capacity to address the mental health needs of this population [4], generating a substantial gap in treatment, especially among vulnerable populations [5].

In response to the limitations of health care, it is essential to provide children and adolescents with resources tailored to strengthening resilience as an essential factor for mental health. Resilience has been defined as the ability to cope with and overcome adversity while maintaining psychological well-being in the face of difficult circumstances [6, 8]; being a set of dynamic and multifactorially based mechanisms, with determinants that are both individual, familial and community-based [9]. From community factors approach the school environment stands out as a key setting for the development of their capacities, with teachers playing a critical role. Therefore, in low- and middle-income countries such as Colombia, school-based mental health interventions have emerged as effective and accessible strategies to promote emotional and behavioral well-being in adolescents, particularly in settings affected by armed conflict [10, 11].

Teachers, alongside families, are key caregivers who support children’s development and emotional well-being, especially in contexts of adversity. In this group, resilience has been associated with improved emotional regulation, greater empathy and ability to assess others’ affective states, as well as enhanced self-efficacy and self-esteem in their professional role [12]. Resilient teachers who demonstrate sensitivity and understanding of their students’ challenges tend to build stronger relationships, which is associated with improved learning outcomes, higher academic performance, better conflict resolution skills, and the parallel development of socioemotional competencies [13, 14]. While some teacher-led resilience interventions have shown positive outcomes in children’s mood and post-traumatic symptoms [15], others report null effects [16]. In Latin America, evidence remains limited. In Colombia, a school-based pilot demonstrated improvements in self-esteem, empathy, and perseverance among both teachers and students [17, 18].

Despite growing recognition of the role teachers play in fostering resilience among students, there is little evidence on how teachers’ social-demographic and socio-emotional characteristics impact their own resilience and how this relates to their ability to support students’ well-being. Resilience among teachers, as in the general population, is a dynamic construct influenced by multiple factors. A review by Zhang and Luo [11] analyzed 22 studies on teacher resilience, highlighting that certain sociodemographic characteristics, such as having a postgraduate degree, being married, and having children, appear to be associated with higher levels of resilience. No gender differences were identified. Teachers who feel motivated in their workplace and benefit from favorable well-being conditions also tend to exhibit greater resilience. On the other hand, lower resilience levels have been correlated with mental health problems such as anxiety, depression, fear, high stress, and occupational burnout. Overall, the evidence suggests that there is a need to understand specifically how interventions with psychosocial impact can affect caregiver resilience, and whether targeting the resilience of caregivers such as teachers can consequently generate positive outcomes for children and adolescents [19].

Positioned in the Colombian context, where schools often operate in settings of high psychosocial vulnerability, the “Conmigo, Contigo y con Todo” (3C) strategy—developed by the Saldarriaga Concha Foundation—has emerged as a structured psychoeducational intervention aimed at promoting resilience, compassion, and mental health. The model draws on cognitive-behavioral therapy and third-generation psychological approaches and is organized into three thematic blocks: *Conmigo* (self-awareness, emotional regulation, self-compassion, sense of life and spirituality), *Contigo* (empathy, compassion, interpersonal relationships, problem-solving), and *Con Todo* (acceptance, creativity, critical thinking, environmental decision-making, and identification of risk and protective factors). These components target the development of socioemotional skills essential for adaptive coping and psychosocial well-being. Evidence from previous implementations of the 3 C model—particularly in family and early childhood settings—suggests that it enhances parental resilience through a multicomponent strategy that integrates everyday contexts and community participation. However, further research is needed to evaluate its impact on teachers’ practices and well-being. In this study, an adapted school-based version was implemented between June 2023 and August 2024 in schools from Boyacá, Vaupés, and Amazonas. Teachers in the intervention group completed a six-month training to act as multiplier agents, delivering the 12-module program across 6–8 sessions with student groups during the school calendar [12].

Addressing mental health challenges among children and adolescents in Colombia, particularly in contexts affected by armed conflict and forced displacement; requires targeted interventions within school environments. Despite this need, a significant gap in the literature: insufficient information or studies have assessed resilience in Colombian teachers exposed to armed conflict using large, regionally diverse samples and standardized assessment tools. This limitation constrains the development and implementation of scalable, evidence-based interventions aimed at strengthening teacher support and promoting school-based mental health strategies.

This study aims to examine the association between sociodemographic variables and resilience, understood as the core capacity for adaptive functioning and emotional recovery; among teachers, prior to the implementation of an intervention designed to enhance socioemotional competencies. The analysis was conducted with participants of the 3C model in the Colombian departments of Vaupés, Boyacá, and Amazonas. The findings seek to contribute empirical evidence to inform the design of psychosocial interventions within school contexts, recognizing teachers as critical agents in the socioemotional development of children and adolescents, and thereby supporting the formulation of evidence-based public mental health policies.

## Objective

To examine the association between sociodemographic and socioemotional factors and resilience levels in teachers prior to their participation in the “Conmigo, Contigo, Con Todo” (3C) program in three Colombian departments affected by armed conflict: Amazonas, Boyacá, and Vaupés.

## Methods

### Participants

The study included teachers from 56 public schools located in the Colombian departments of Vaupés, Boyacá, and Amazonas, who were hired to work in these institutions during the 2023–2024 academic years and taught students from second to eleventh grade. Eligible participants were required to be Spanish-speaking and actively employed at the selected schools at the time of data collection; teachers who did not wish to participate were excluded. Data were collected prior to the start of the training phase of the *Conmigo*, *Contigo y con Todo* (3C) program.

A stratified sampling strategy was used, with departments defined as strata, based on the territorial scope of the 3 C program implementation [20]. The selected departments were prioritized because they are designated as Development Programs with a Territorial Approach (PDET), which are regions identified by the Colombian government as requiring urgent social and institutional strengthening within the framework of the territorial peace strategy established after the Final Peace Agreement with the FARC-EP.

The distribution of the sample reflects the number of public schools included in each department: Of the 56 participating institutions, 36 were located in Boyacá, 10 in Amazonas, and 10 in Vaupés, resulting in a sample distribution of 451 teachers from Boyacá (65.1%), 129 from Amazonas (18.6%), and 113 from Vaupés (16.3%), proportional to the number of schools per stratum. Accordingly, a larger proportion of teachers were recruited from Boyacá. Territorial coverage also differed across departments, as the intervention was implemented in all three municipalities of Vaupés, in four of the five municipalities of Amazonas, and in 21 of the 123 municipalities of Boyacá. Thus, differences in sample size across departments are consistent with the stratified design and reflect the operational scale and geographic coverage of the intervention rather than unequal sampling procedures.

The final sample comprised 693 teachers with a mean age of 46.9 years (SD = 10.2). The majority were female (66.2%) and resided in urban areas. Most participants belonged to a low socioeconomic stratum, particularly in Amazonas (89.1%) and Vaupés (97.4%), while in Boyacá the distribution was more balanced between low and middle strata. A low prevalence of exposure to traumatic events was reported overall, including armed conflict (8.8%) and natural disasters (1.4%). The proportion of teachers reporting victimization by armed conflict was markedly higher in Vaupés (43.0%) compared to Amazonas (6.3%) and Boyacá (0.9%). None of the participants identified as belonging to an ethnic group in Boyacá, whereas a majority of those in Vaupés identified as Indigenous (76.3%). Detailed sociodemographic characteristics stratified by department are presented in Table 1.

### Data collection

#### Sociodemographic characteristics

Data collection was carried out prior to the start of teacher training in the 3C model. Sociodemographic data were collected for each participant, through a self-administered questionnaire, including age, sex, socioeconomic status, the presence of some type of disability and ethnicity. The location of their homes, differentiated between rural and urban areas, were included.Exposure to natural disasters and armed conflict was determined based on each participant’s perception, without specified time criteria, using a simple dichotomous (yes/no) response. Furthermore, the level of satisfaction with family relationships and the support perceived by their family, the relationship with their partner, in addition to the role they perceive they play in their home.

To minimize the potential impact of cultural and ethnic diversity on instrument comprehension, all instruments were self-administered with trained personnel present throughout data collection to clarify doubts and ensure adequate understanding of the items. Field personnel received prior training in cultural sensitivity. Standardized instructions were provided in Spanish, the official language of instruction in all participating schools, including those in departments with a predominantly Indigenous population such as Vaupés and Amazonas.

#### Standardized instruments

All participants completed standardized instruments to assess levels of resilience, compassion, and prosocial behavior, as well as mental health screening scales of anxiety, depression, and post-traumatic stress. These instruments were self-administered, and participants received support if they had any doubts or needed clarification about the questions. The collected data were processed using REDCap data management tools.

**Connor-Davidson Resilience Scale; CD-RISC**

The Connor-Davidson Resilience Scale (CD-RISC) is a 25-item self-report instrument designed to assess psychological resilience across multiple domains. Items are rated on a Likert scale from 0 (“not true at all”) to 4 (“true nearly all the time”), yielding a total score ranging from 0 to 100, with higher scores indicating greater perceived resilience [21]. The scale was originally developed and validated in adult populations and has demonstrated good psychometric properties, including high internal consistency and convergent validity with measures of stress, disability, and social support [21]. In Spanish-speaking contexts, several studies have reported adequate reliability and construct validity of the CD-RISC [22].

In Colombia, the CD-RISC has been formally validated in vulnerable adolescent populations, demonstrating a one-factor structure and good internal consistency (Cronbach’s α = 0.86) [23]. Although no specific psychometric validation has been conducted in Colombian teachers, the scale has been used in adult Colombian samples exposed to armed conflict. Notably, the CD-RISC was employed as a primary outcome measure in the evaluation of the “Conmigo, Contigo y con Todo” (3 C) program for caregivers of young children, where it demonstrated sensitivity to change in adult participants [12].

**Compassion Scale; ECOM**

The Compassion Scale (ECOM) is a 10-item self-report instrument designed to assess compassion as a multidimensional construct, defined as the sensitivity to one’s own and others’ suffering, recognition of suffering as a shared human experience, and motivation to alleviate that suffering. Items are rated on a 5-point Likert scale, with higher scores indicating greater perceived compassion [24]. The scale was originally developed and validated in a Spanish-speaking population in Mexico. Psychometric evaluation demonstrated a unidimensional factor structure and good construct validity, with significant associations observed between compassion, emotional regulation, and psychological well-being. The instrument also showed high internal consistency (Cronbach’s α = 0.89) in the original validation study [24].

Although no formal cultural adaptation or validation study of the ECOM has been conducted in Colombia, the scale was selected for the present study due to its conceptual relevance, its original development in Spanish, and its applicability to psychosocial and educational contexts.

**Prosocial battery; PSB**

The Prosocial Personality Battery (PSB) is a self-report instrument originally developed to assess stable prosocial personality traits in adults, including empathy-oriented concern and the tendency to engage in helping behaviors [25]. The present study used the brief version of the PSB. A Spanish-language version validated in Mexican university students, reporting acceptable internal consistency (α = 0.71) and supporting a two-factor structure reflecting empathy-oriented prosociality and availability to help [26]. Their findings also highlight the need to evaluate the PSB through internal consistency analyses when applied in new cultural contexts.

**Hamilton Anxiety Rating Scale; HARS**

The Hamilton Anxiety Rating Scale (HARS) is a clinician-rated instrument widely used to assess the severity of anxiety symptoms. It consists of 14 items covering both psychological and somatic manifestations of anxiety, each rated on a scale from 0 (not present) to 4 (very severe), yielding a total score ranging from 0 to 56 [27]. The HARS has been validated in Spanish-speaking populations, demonstrating psychometric properties comparable to those of the original version, including adequate reliability and construct validity [28]. Due to its extensive use in Latin American clinical and research contexts, the Spanish version of the HARS was considered appropriate for use in this study. Although no specific cultural adaptation of the HARS has been conducted for Colombian teachers, the scale was applied as a screening tool to capture anxiety symptom severity rather than to establish clinical diagnoses. Participants received standardized instructions and support during administration to ensure comprehension of the items.

**Whooley depression screen**

This is a simple and brief screening tool for detecting cases of depression, consisting of two questions that inquire about depressed mood and anhedonia. Affirmative responses to both questions yield a sensitivity of 96% (95% CI: 90–99%) and a specificity of 57% (95% CI: 53–62%) [29]. These questions are recommended as a screening tool in the Colombian clinical practice guidelines for depression [30].

**Posttraumatic Stress Checklist-Civilian version; PCL-C**

The Posttraumatic Stress Disorder Checklist–Civilian Version (PCL-C) is a 17-item self-report instrument designed to assess symptoms associated with posttraumatic stress disorder (PTSD) in civilian populations. Items correspond to DSM-IV PTSD symptom clusters and are rated on a five-point Likert scale ranging from 1 (not at all) to 5 (extremely), yielding a total score that reflects symptom severity [31]. The PCL-C has demonstrated good psychometric properties in adult populations, including high internal consistency, test–retest reliability, and convergent validity with clinical diagnoses of PTSD. 

Spanish versions of the PCL-C have been validated in different Latin American and Spanish-speaking populations, reporting adequate reliability coefficients (Cronbach’s α typically > 0.85) and supporting its use as a screening instrument for PTSD symptoms. In Colombia, the PCL-C has been employed in large-scale epidemiological research, including the National Mental Health Survey, supporting its applicability in the local context [57]. Although no specific cultural adaptation has been conducted for Colombian teachers, the instrument was used in this study as a screening tool rather than for diagnostic purposes

To address the limitations inherent to the lack of prior cross-cultural adaptation of the instruments to the local context, and with the objective of ensuring the methodological validity of the measurements, the psychometric properties were evaluated using data from the present study’s sample. Specifically, the internal consistency of each scale was estimated by calculating Cronbach’s alpha coefficient on the individual items, obtaining point estimates and their respective 95% confidence intervals. The results confirmed high reliability within the study population. For the CD-RISC scale, an alpha = 0.958 (95% CI: 0.954–0.963) was obtained; for the ECOM scale, an alpha = 0.934 (95% CI: 0.927–0.941); and for the PSB scale, an alpha = 0.831 (95% CI: 0.813–0.849). Likewise, the PCL-C scale demonstrated excellent consistency with an alpha = 0.952 (95% CI: 0.947–0.957). Regarding the anxiety and depression screenings, the Hamilton Anxiety Rating Scale (HARS) registered an alpha = 0.925 (95% CI: 0.917–0.934), and the Whooley depression screening tool showed an alpha = 0.809 (95% CI: 0.779–0.836). These psychometric findings demonstrate that, regardless of specific cultural validation, the instruments were highly reliable and consistent for data collection and the structuring of epidemiological analyses in this research

### Data analysis

In the data analysis phase, the internal consistency of each clinical scale was first estimated by calculating Cronbach’s alpha coefficient on the individual items, obtaining point estimates and their respective 95% confidence intervals [32]. Subsequently, to determine the appropriateness of using parametric or non-parametric tests in the bivariate and comparative analyses, the distribution of the total scores for each instrument was evaluated using the Shapiro-Wilk normality test [33]. Hypothesis testing and comparison of medians between independent groups were conducted using the Kruskal-Wallis test [34] and ANOVA where appropriate [35].

To classify continuous variables into severity categories or risk levels presented in the analysis, the cut-off points established in the original manuals and international validation literature for each instrument were adopted. Specifically, for the HARS, severity was stratified into: mild anxiety (0–17 points), mild to moderate (18–24 points), moderate to severe (25–30 points), and very severe anxiety (31–56 points) [27]. In the case of the PCL-C, a score of 30 points was defined as a positive indicator for possible post-traumatic stress disorder, based on evidence reporting 82% sensitivity and 76% specificity at this threshold [36]. For depression screening using the Whooley questions, a positive result required at least one affirmative response, in accordance with the recommendations of the Colombian Clinical Practice Guidelines and the original development report of the instrument [29, 30]. In contrast, instruments without standardized severity cut-off points, such as the CD-RISC and the ECOM, were analyzed as continuous variables.

A descriptive analysis was conducted for the sociodemographic, emotional, and resilience information of the teachers, stratified by department of the educational institution to which they belonged. Qualitative variables were described as absolute and relative frequencies, while quantitative variables were described as means with standard deviation or medians with their respective interquartile range.

The normality of quantitative variables was assessed using the Shapiro-Wilk test, complemented by visual inspection of histograms. The findings evidenced that the scores of the CD-RISC, ECOM, PCL-C, HARS, and Whooley scales did not follow a normal distribution (*p* < 0.05), whereas only the PSB scale showed a normal distribution (*p* = 0.130), justifying the differential use of non-parametric and parametric comparative tests across analyses.

For the construction of the multivariable linear regression model, a purposeful selection strategy complemented by theoretical and clinical criteria was employed. Initially, all variables that demonstrated a p-value < 0.50 in the bivariate analysis were included as candidates [37]. Additionally, variables considered universal confounders or of a priori clinical importance were forced into the model regardless of their initial level of statistical significance [38]. Specifically, sociodemographic factors such as age and sex were retained as mandatory adjustment variables given their established relevance in the Colombian epidemiological context [39]. Subsequently, the model was manually refined by sequentially removing variables that did not maintain *p* < 0.05, verifying at each step that the exclusion did not alter the coefficients of the remaining variables by more than 10%, thereby guaranteeing adequate control of potential confounding effects in the final model.

To ensure the validity of the regression analysis, post-estimation diagnostic procedures were performed. Multicollinearity was evaluated using the Variance Inflation Factor (VIF), yielding a mean VIF of 1.21 and a maximum VIF of 1.320, both well below the critical threshold, confirming the total absence of severe collinearity among the independent variables. Notably, although compassion (ECOM) and prosociality (PSB) are conceptually related constructs, their correlation was moderate (Spearman rho = 0.41) and VIF values confirmed they could be included simultaneously without redundancy. A multivariate linear regression model was then fitted to estimate the independent associations of selected variables with resilience scores. A sensitivity analysis was performed to assess the potential influence of outliers: extreme values were identified using the interquartile range criterion, and models were re-estimated excluding these observations. Additionally, Cook’s distance and studentized residuals were examined to detect influential cases. Results confirmed that the direction and significance of main associations remained stable, and no individual observations exerted disproportionate influence on model estimates. All analyses were performed in Stata 16.0.

## Results

### Descriptive analysis

#### Sociodemographic characteristics

Regarding exposure to armed conflict, important regional differences were observed. While the overall prevalence of teachers reporting having been victims of armed conflict was relatively low (8.8%), this proportion was substantially higher in Vaupés, where 42.98% of participants reported exposure, compared to much lower percentages in Amazonas and Boyacá. This marked difference reflects the historical intensity of armed conflict in Vaupés and provides relevant contextual information for interpreting the mental health and resilience profiles observed in this department.

**Table 1:** Sociodemographic characteristics of the total population and departments, *n* = 693

	Total	Amazonas	Boyacá	Vaupés	*p*- value
	*n* = 693	*n* = 129	*n* = 451	*n* = 113	
	% (*n*)	% (*n*)	% (*n*)	% (*n*)	
Age (years), average and SD	47.13 ± 9.91	44.04 ± 9.43	48.80 ± 9.25	44.04 ± 11.39	< 0.001†
Sex					
Female	457 (65.95)	66 (51.56)	342 (75.83)	49 (42.98)	0.001*
Male	236 (34.05)	62 (48.44)	109 (24.17)	65 (57.02)	
Housing location zone					
Urban	547 (78.93)	75 (58.59)	411 (91.13)	61 (53.51)	0.001*
Rural	147 (21.07)	53 (41.41)	40 (8.87)	53 (46.49)	
Marital status					
Single	173 (24.96)	32 (25.00)	123 (27.27)	18 (15.79)	
Married	270 (38.96)	30 (23.44)	209 (46.34)	31 (27.19)	
Cohabiting	182 (26.26)	57 (44.53)	65 (14.41)	60 (52.63)	
Separated	29 (4.18)	8 (6.25)	16 (3.55)	5 (4.34)	0.001*
Divorce	29 (4.18)	0 (0.00)	29 (6.43)	0 (0.00)	
Widowed	10 (1.44)	1 (0.78)	9 (2.00)	0 (0.00)	
Socioeconomic Status					
Low (1–2)	434 (62.63)	114 (89.06)	208 (46.34)	111 (97.37)	
Middle (3–4)	208 (35.79)	14 (10.94)	231 (51.22)	3 (2.62)	0.001*
High (5–7)	11 (1.59)	0 (0.00)	11 (2.44)	0 (0.00)	
Ethnicity					
Not recognized in any	507 (73.16)	49 (38.28)	443( 98.23)	15 (13.16)	
Indigenous	157 (22.66)	67 (52.34)	3 (0.67)	87 (76.32)	0.001*
Afro-Colombian and others (Raizal, Palenquero)	29 (4.18)	12 (9.38)	5 (1.11)	12 (10.53)	
Victim of natural disasters					
No	683 (98.56)	124 (96.98)	449 (99.49)	110 (96.49)	0.010*
Yes	10 (1.44)	4 (3.13)	2 (0.44)	4 (3.51)	
Victim of armed conflict					
No	632 (91.20)	120 (93.75)	447 (99.11)	64 (56.64)	0.001*
Yes	61 (8.80)	8 (6.25)	4 (0.89)	49 (42.98)	
Disability condition					
No	688 (99.28)	127 (99.22)	450 (99.78)	111 (97.37)	
Yes	5 (0.72)	1 (0.78)	1 (0.22)	3 (2.63)	0.025*
Family support in the event of problems					
No	10 (1.44)	4 (3.13)	5 (1.11)	1 (0.88)	
Yes	683 (98.56)	124 (96.88)	446 (98.89)	113 (99.12)	0.206*
Satisfaction with family relationships					
Dissatisfied	15 (2.16)	6 (4.69)	4 (0.89)	5 (4.39)	0.007*
Satisfied	678 (97.84)	122 (95.31)	447 (99.11)	109 (95.61)	
Relationship with partner					
Dissatisfied	18 (3.70)	4 (4.40)	11 (3.62)	3 (3.26)	
Satisfied	469 (96.30)	87 (95.60)	293 (96.38)	96.74 (89)	0.914*
Role at household					
Decisions made in the household are made, but not consulted	13 (1.88)	4 (3.13)	4 (0.89)	5 (4.39)	
Decisions that are made at household, but are consulted	120 (17.32)	14 (10.94)	93 (20.62)	13 (11.40)	
Head of household	523 (75.47)	100 (78.13)	337 (74.72)	86 (75.44)	0.001*
Provider	37 (5.34)	10 (7.81)	17 (3.77)	10 (8.77)	

*SD* standard deviation, *IQR* interquartile range. †Kruskal-Wallis test (continuous variables); *Pearson’s Chi-square test (categorical variables). Absolute and relative frequencies are presented for categorical variables

### Findings of the assessment tools

**Fig. 1:**
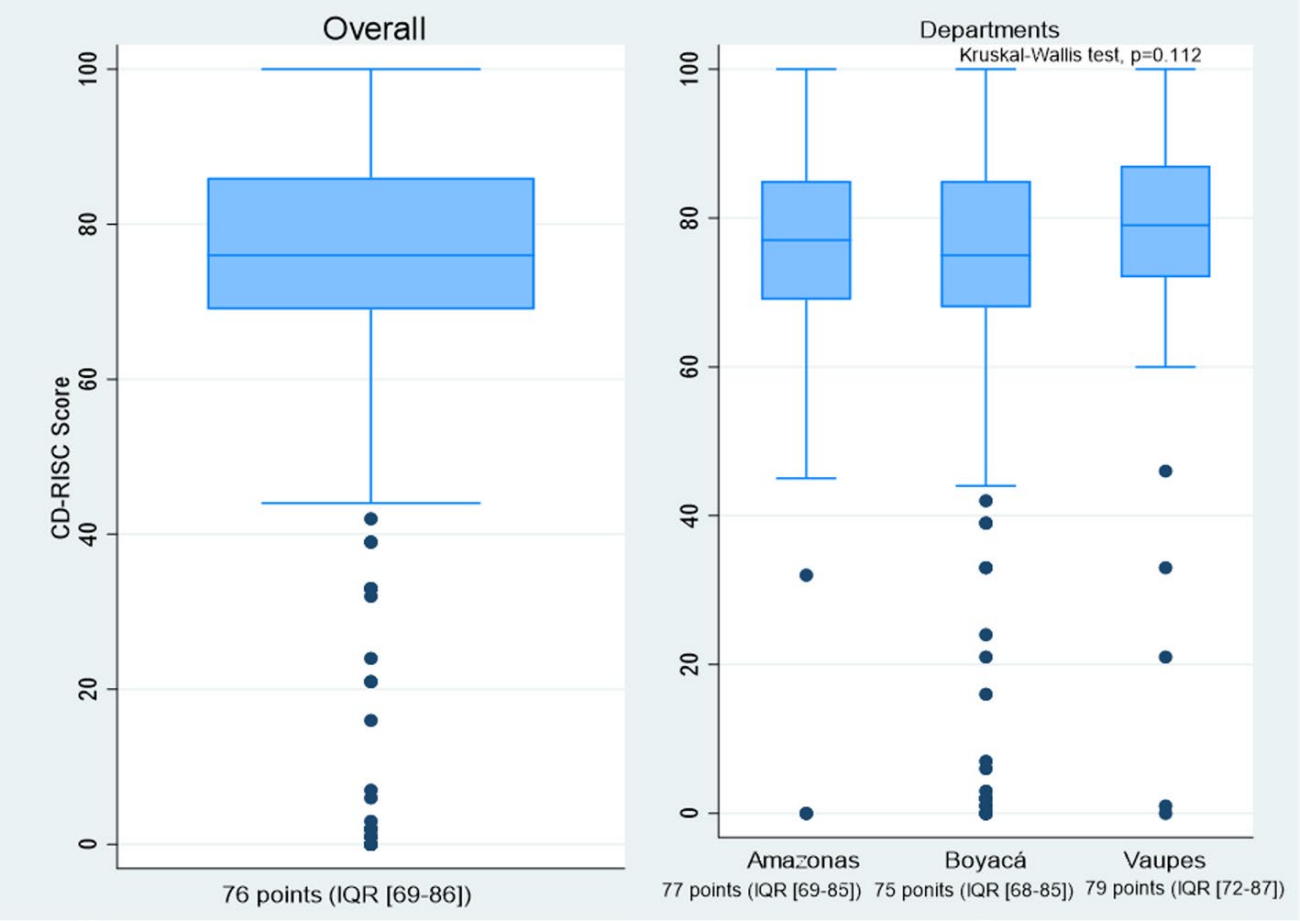
CD-RISC resilience scores by total sample and department

The median resilience score for the entire sample was 76 points (IQR [69–86]). Higher median scores were observed among teachers from Vaupés, whereas lower scores were found among teachers from Boyacá (Fig. 1).

Regarding compassion, assessed using the ECOM scale, the median score was 69 points, with Vaupés again showing the highest values. In contrast, prosociality measured by the PSB scale was highest in Boyacá (111.85 ± 13.80), slightly above the overall mean (110.12 ± 13.50).

With respect to mental health indicators, Vaupés showed a higher frequency of positive screenings for depression (45.61%) and higher anxiety scores on the HARS scale (median 9.0 [4–16]). In the total sample, 41.27% screened positive for depression and the median anxiety score was 8.0 [IQR: 3–14]. The median post-traumatic stress score was 25.0 [19.0–33.0], with 35.06% of participants screening positive, particularly in Amazonas and Vaupés (Table 2). Overall, these findings provide a general overview of the population’s health profile, with no statistically significant differences observed across departments in mental health screening outcomes.

**Table 2:** Compassion, prosocial behavior and mental health screening scores at baseline

	Total *n* = 693 % (*n*)	Amazonas *n* = 128 % (*n*)	Boyacá *n* = 451 % (*n*)	Vaupés *n* = 114	*p* -value
Compassion (ECOM), Median[Q1-Q3]	69 [63–76]	69 [63–76]	69 [62–76]	71 [63–77]	0.619*
Prosocial Personality Battery (PSB), average and standard deviation	110.12 ± 13.50	107.41 ± 11.63	111.85 ± 13.80	106.29 ± 13.02	< 0.001**
Post-Traumatic Stress Checklist (PCL-C), Median [Q1-Q3]	25.0 [19.0–33.0]	27.5 [20–35]	27.0 [20–35]	23.0 [19.0–32.0]	0.001*
PTSD Screening					
Negative Stress	448 (64.94)	70 (54.69)	314 (69.62)	65 (57.02)	0.001†
Positive Stress	245 (35.5)	59 (45.74)	137 (30.38)	49 (42.98)	
Anxiety (Hamilton Anxiety Rating Scale), Median [Q1-Q3]	8.0 [3–14]	8.0 [3-15.5]	7.0 [3–13]	9.0 [4–16]	0.130*
Anxiety level					
Mild	568 (81.96)	107 (83.59)	372 (82.48)	89 (78.07)	
Mild to moderate	78 (11.26)	17 (13.28)	41 (9.09)	20 (17.54)	0.041†
Moderate to severe	36 (5.19)	3 (2.34)	28 (6.21)	5 (4.39)	
Very severe	11 (1.59)	1 (0.78)	10 (2.22)	0 (0.00)	
Depression (Whooley Test)					
Negative	407 (58.73)	77 (60.16)	268(59.42)	62 (54.39)	0.581 †
Positive	286 (41.27)	51 (39.84)	183(40.58)	52 (45.61)	

For quantitative variables, median [Q1–Q3] is reported for non-normally distributed variables (CD-RISC, ECOM, HARS, PCL-C), while mean ± SD is reported for the normally distributed variable (PSB). Normality was assessed using the Shapiro-Wilk test. Absolute and relative frequencies are presented for categorical variables. *Kruskal-Wallis test; **ANOVA F-test; †Pearson’s Chi-square test. Abbreviations: CD-RISC: Connor-Davidson Resilience Scale; ECOM: Compassion Scale; PSB: Prosocial Personality Battery; HARS: Hamilton Anxiety Rating Scale; PCL-C: Post-Traumatic Stress Checklist - Civilian version; PTSD: Post-Traumatic Stress Disorder

### Bivariate correlations

Spearman correlations were computed to examine bivariate associations between resilience and socioemotional variables. Resilience (CD-RISC) showed positive correlations with compassion (ECOM: rho = 0.305; 95% CI: 0.236–0.371; *p* < 0.001) and prosociality (PSB: rho = 0.322; 95% CI: 0.254–0.387; *p* < 0.001). Conversely, resilience was negatively correlated with anxiety (HARS: rho = − 0.307; 95% CI: −0.373 to −0.238; *p* < 0.001), post-traumatic stress symptoms (PCL-C: rho = − 0.278; 95% CI: −0.345 to −0.207; *p* < 0.001), and positive depression screen (Whooley: rho = − 0.224; 95% CI: −0.293 to −0.152; *p* < 0.001). Among socioemotional variables, the highest correlation was observed between anxiety and post-traumatic stress symptoms (HARS–PCL-C: rho = 0.715; 95% CI: 0.676–0.749; *p* < 0.001), reflecting the known clinical overlap between these constructs. The association between compassion and depression screening did not reach statistical significance (ECOM–Whooley: rho = − 0.065; 95% CI: −0.139 to 0.009; *p* = 0.085). The complete correlation matrix with 95% confidence intervals is presented in Table S1. anxiety (HARS: rho = − 0.307; 95% CI: −0.373 to −0.238; *p* < 0.001), post-traumatic stress symptoms (PCL-C: rho = − 0.278; 95% CI: −0.345 to −0.207; *p* < 0.001), and positive depression screen (Whooley: rho = − 0.224; 95% CI: −0.293 to −0.152; *p* < 0.001). Among socioemotional variables, the highest correlation was observed between anxiety and post-traumatic stress symptoms (HARS–PCL-C: rho = 0.715; 95% CI: 0.676–0.749; *p* < 0.001), reflecting the known clinical overlap between these constructs. The association between compassion and depression screening did not reach statistical significance (ECOM–Whooley: rho = − 0.065; 95% CI: −0.139 to 0.009; *p* = 0.085). The complete correlation matrix with 95% confidence intervals is presented in Table S1.

### Multivariate linear regression

A multivariate linear regression model was fitted to identify factors independently associated with resilience, adjusting for age and sex (Table 3). Post-estimation diagnostics confirmed the validity of the model assumptions. Multicollinearity analysis using the Variance Inflation Factor (VIF) ruled out redundancy among the independent variables, yielding a mean VIF of 1.21 and a maximum VIF of 1.320, both well below the critical threshold, confirming the absence of severe collinearity. The model explained 17.5% of the variance in resilience scores (R^2^ = 0.175, adjusted R^2^ = 0.168; F(6, 686) = 24.32, *p* < 0.001). Higher compassion scores (ECOM: β = 0.23, 95% CI: 0.10–0.36, *p* < 0.001) and higher prosociality scores (PSB: β = 0.43, 95% CI: 0.31–0.54, *p* < 0.001) were significantly associated with increased resilience. Conversely, higher anxiety levels were associated with lower resilience (HARS: β = −0.22, 95% CI: −0.39 to − 0.06, *p* = 0.007). Depression screening status showed a negative association that did not reach statistical significance (β = −2.46, 95% CI: −5.18 to 0.25, *p* = 0.075). Age and sex were not significantly associated with resilience in the adjusted model.

**Table 3:** Multivariate linear regression: factors associated with teacher resilience (CD-RISC)

Variable	β	95% CI	*p*-value
Age (years)	0.01	−0.10, 0.13	0.811
Sex (female vs. male)	1.34	−1.19, 3.87	0.298
Compassion (ECOM)	0.23	0.10, 0.36	< 0.001
Prosociality (PSB)	0.43	0.31, 0.54	< 0.001
Anxiety (HARS)	−0.22	−0.39, − 0.06	0.007
Depression screen positive	−2.46	−5.18, 0.25	0.075

*n* = 693. R^2^ = 0.175, Adjusted R^2^ = 0.168. β = unstandardized regression coefficient; CI = confidence interval. Reference category for sex: male. Age and sex were included as mandatory adjustment variables based on a priori clinical and epidemiological criteria, regardless of their *p*-value in the bivariate screening.

**Fig. 2:**
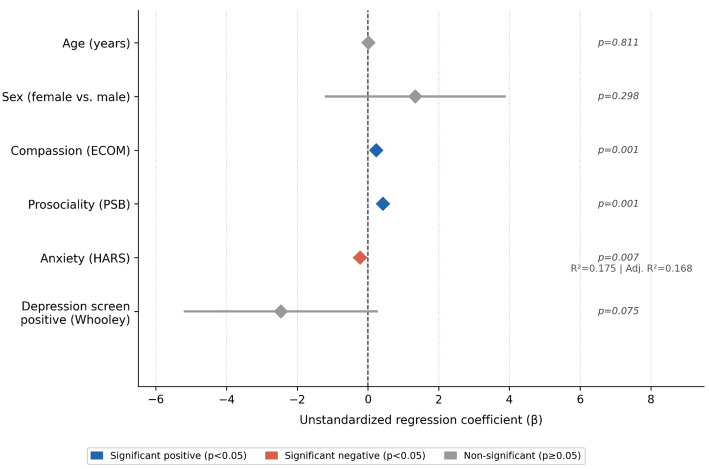
Factors associated with teacher resilience (age- and sex-adjusted multivariate linear regression, *n* = 693). ECOM: Compassion Scale. PSB: Prosocial Personality Battery. HARS: Hamilton Anxiety Rating Scale. Whooley: two-item depression screening test. Diamonds represent unstandardized regression coefficients (β); horizontal lines represent 95% confidence intervals. Blue: significant positive association (*p* < 0.05); Red: significant negative association (*p* < 0.05); Grey: non-significant (*p* ≥ 0.05). Reference category for sex: male

## Discussion

The present study draws on a large sample of teachers in Colombia, examining levels of resilience, compassion, prosociality, and screenings for depression, anxiety, and post-traumatic stress. Moreover, the sample is diverse, as it was drawn from three departments with distinct characteristics, despite all being regions selected within the PDET framework and historically impacted by the armed conflict. The large sample size also guarantees sufficient statistical power to explore these associations using teachers’ resilience scores [1, 11].

The analysis of data from 693 teachers revealed a consistent association between higher levels of compassion and prosociality and increased resilience. On the contrary, elevated anxiety levels and positive depression screening were independently associated with lower resilience scores in the multivariable model, while post-traumatic stress symptoms showed a significant bivariate correlation with resilience but were not retained in the final model. Additionally, the role each teacher holds within their household showed a trend of potential relevance among the sociodemographic variables examined. Although the differences were not statistically significant, higher levels of resilience were observed among those who identified as heads of household and those who reported participating in decision-making, compared to those who identified primarily as providers [40, 41].

Of particular note is the prevalence of positive depression screenings, followed by positive screenings for PTSD. While the proportion of positive cases did not exceed that of negative screenings, these findings remain noteworthy. In Colombia, few studies have specifically assessed the mental health of teachers. Quintero Idárraga & Hernández Calle [42], for example, in a sample of 122 public school teachers from Envigado (Antioquia), reported that 65.8% exhibited symptoms of depression. Furthermore, their multivariate analysis found that the emotional exhaustion component of burnout significantly increased depressive symptoms among teachers (OR = 4.0, CI = 1.67–9.58). Similarly, Guevara‑Manrique et al. [43] found that 29.5% of teachers in a school in the Cauca department—a region with high levels of violence—met criteria for mental health conditions, with depressive symptoms being the fourth most frequently reported issue.

Regarding PTSD, no specific data were identified for this population group. This represents a critical gap: there are no diagnostic studies on teachers’ mental health based on large, representative population samples in Colombia. Additionally, the variability in measurement instruments used across existing studies limits comparability and the ability to draw broader conclusions [10, 11]. Therefore, the present study may serve as a foundation for future research with larger and more representative samples of teachers.

Among the key findings, the positive correlation between resilience and compassion was particularly noteworthy. Although the strength of the association was weak, it is consistent with existing literature, which has documented a positive relationship between self-compassion, resilience, and overall well-being [44]. Previous studies have demonstrated that both compassion and resilience can enhance individuals’ capacity to manage stress and increase job satisfaction [45]. Accordingly, interventions aimed at fostering self-compassion have been identified as having significant potential to strengthen teacher resilience, especially in demanding educational settings located in socioeconomically disadvantaged contexts [46], such as the Colombian departments selected for this study.

In contrast, the association between compassion and positive depression screening did not reach statistical significance (ECOM–Whooley: rho = − 0.065; 95% CI: −0.139 to 0.009; *p* = 0.085). This null finding may reflect the binary and limited nature of the Whooley screen as a depression measure—capturing only case-level presence or absence—which may lack the sensitivity to detect the more nuanced relationship between compassion and subclinical depressive symptoms. Future studies using continuous depression measures would allow a more precise examination of this association.

These empirical associations are consistent with the theoretical frameworks underlying the instruments used in this study. Connor and Davidson conceptualized resilience as a dynamic, multidimensional capacity for adaptive coping that can be strengthened through positive social and emotional resources [21]. From this perspective, the positive associations observed with compassion and prosociality are theoretically coherent: López Tello and Moreno Coutiño defined compassion as sensitivity to one’s own and others’ suffering combined with a motivation to alleviate it [24], a disposition that may reinforce the coping and recovery processes central to resilience. Similarly, Penner et al. described prosocial personality traits as stable orientations toward empathy and helping behavior [25], which may buffer against the erosive effects of chronic adversity on psychological well-being. Conversely, the inverse associations with anxiety and post-traumatic stress are aligned with Hamilton’s original conceptualization of anxiety as a clinically significant interference with adaptive functioning [27], and with the PCL-C framework, which captures the symptomatic burden that impairs recovery and coping capacity in trauma-exposed populations [31].

Family dynamics represent a contextual factor that should be considered when implementing programs aimed at fostering resilient environments. Although no statistically significant relationship was found in the present study between satisfaction with family or partner relationships and resilience levels, the overall sample reported high levels of family satisfaction. Existing literature has identified strong family relationships, characterized by joint problem-solving and mutual support, as a key protective factor that enhances teachers’ resilience [40, 41]. This aligns with findings from the present study, in which participants who identified as heads of household or reported being consulted on family matters tended to show higher resilience levels, even though these associations did not reach statistical significance.

The negative relationship observed between resilience and symptoms of anxiety and post-traumatic stress underscores the importance of addressing teachers’ mental health. Similar associations have been reported in previous studies, which also suggest that this correlation is linked to perceived vulnerability to mental illness [47]. Higher levels of resilience have been identified as a protective factor against the development of mental health disorders, particularly due to their role in enhancing individuals’ capacity to cope with trauma. This protective effect has been especially emphasized in contexts marked by extreme adversity, such as war-related situations [48, 49]. Notably, the strong correlation observed between anxiety and post-traumatic stress symptoms (HARS–PCL-C: rho = 0.715; 95% CI: 0.676–0.749) is consistent with the well-documented clinical overlap between these constructs, particularly in populations exposed to chronic adversity and armed conflict. This finding suggests that in contexts such as Vaupés and Amazonas, anxiety and PTSD symptomatology may represent overlapping dimensions of a broader trauma response rather than fully independent constructs, which has implications for the design of targeted mental health interventions in these settings.

In the Colombian context, Maldonado-Carreño et al. [50] demonstrated that positive and responsive interactions between teachers and students are significantly associated with children’s developmental outcomes. These findings underscore the need to address teachers’ mental health, given the relationship between their well-being and the quality of teacher–student interactions. Teachers experiencing mental health difficulties, such as stress, burnout, or depression, may negatively influence students’ academic performance [51]. Positive relationships with students have been identified as a protective factor against the effects of adversity in children. Moreover, teachers play a fundamental role in students’ psychosocial development, contributing to improvements in self-concept, academic adaptability, and socioemotional skills [52, 53].

The coexistence of high resilience and elevated depressive symptoms observed in Vaupés should be interpreted with caution. Rather than representing a contradiction, this finding is approached here as an interpretive and exploratory hypothesis, particularly in indigenous and intercultural contexts. Prior research has shown that psychological constructs such as resilience, depression, and prosociality — largely developed within Western epistemological frameworks—may not fully capture culturally grounded meanings of wellbeing, suffering, and adaptation in indigenous populations [54]. Consequently, elevated scores on standardized scales may coexist with culturally embedded coping strategies and collective resilience processes that are not adequately reflected by these instruments. This interpretation is consistent with the literature on cultural validity, which emphasizes the need to critically examine the cross-cultural applicability of psychometric tools and to avoid assuming conceptual equivalence across sociocultural contexts [54, 55].

The reported findings, together with existing scientific evidence, highlight the need to address teachers’ mental health through promotional and preventive interventions such as the 3 C model. This model, grounded in a theory of change described by González Ballesteros et al. [12], aims to strengthen resilience by targeting risk factors at multiple levels: macro level (effects of armed conflict and extreme poverty), meso level (community violence and barriers to socioemotional and psychosocial support and education), and micro level (maladaptive caregiving practices and poor caregiver well-being).

These findings contribute to the growing body of evidence supporting the value of comprehensive interventions within educational settings. This is particularly relevant in low- and middle-income countries such as Colombia, where the number of specialized professionals and trained general personnel in mental health remains markedly insufficient, limiting the capacity to respond to the needs of children and adolescents [4]. In this context, teacher-centered strategies not only offer a feasible and scalable approach to promoting mental health, but may also help reduce the significant treatment gap for mental disorders in young populations [5, 19].

### Limitations

This study presents several limitations. As a cross-sectional design was employed, it is not possible to establish causal relationships between the examined factors and resilience scores; thus, longitudinal studies are warranted. Additionally, the data were self-reported by the teachers, which may introduce courtesy bias. Moreover, although measures were taken during data collection to ensure comprehension of the instruments across cultural contexts, the absence of formal cross-cultural adaptation of the scales for Indigenous populations in Amazonas and Vaupés represents a methodological limitation that restricts the comparability of scores across departments and the generalizability of findings to these communities. Lastly, the exclusion of key dimensions of resilience—such as community resilience and coping strategies—restricts a comprehensive understanding of resilience as a social phenomenon within the communities where the study was conducted.

## Conclusion

The findings presented in this study offer valuable insights for the development and adaptation of resilience-based mental health promotion and prevention interventions in Colombia and in educational settings, as well as for public policymaking, particularly in light of the recent mandatory implementation of socio-emotional education models at all educational levels [56]. In this regard, such adaptations should also include a cultural tailoring component to ensure that strategies and measurement tools are appropriate and meaningful across the diverse contexts and worldviews present in the country. Mental health programs implemented in educational settings should prioritize teacher mental health, recognizing their well-being as a key factor in positively influencing student outcomes. Furthermore, this study lays the groundwork for future research aimed at evaluating the long-term impact of the 3 C program using longitudinal designs.

## Electronic Supplementary Material

Below is the link to the electronic supplementary material.

Supplementary Material 1

## Data Availability

The datasets generated and analyzed during this study are not publicly available to protect participant confidentiality in accordance with ethical approval conditions. De-identified data are available from the corresponding author upon reasonable request and with appropriate ethical permissions.
