# Supplementary material for: Socioemotional factors associated with teacher resilience in Colombian communities affected by armed conflict a cross-sectional study
Source: Discov Ment Health. 2026 May 22;6(1):94. doi: 10.1007/s44192-026-00449-w (PMC13221519; doi:10.1007/s44192-026-00449-w)
Supplement: Supplementary file 1 — Supplementary Material 1 [file 44192_2026_449_MOESM1_ESM.docx]

**Table S1. Spearman correlation matrix between resilience and socioemotional variables (n = 693)**

| **Variable 1** | **Variable 2** | **rho** | **95% CI** | **p-value** |
| --- | --- | --- | --- | --- |
| CD-RISC | ECOM | 0.305 | 0.236 – 0.371 | <0.001 |
| CD-RISC | PSB | 0.322 | 0.254 – 0.387 | <0.001 |
| CD-RISC | HARS | −0.307 | −0.373 – −0.238 | <0.001 |
| CD-RISC | PCL-C | −0.278 | −0.345 – −0.207 | <0.001 |
| CD-RISC | Whooley | −0.224 | −0.293 – −0.152 | <0.001 |
| ECOM | PSB | 0.408 | 0.344 – 0.468 | <0.001 |
| ECOM | HARS | −0.085 | −0.159 – −0.011 | 0.024 |
| ECOM | PCL-C | −0.120 | −0.193 – −0.046 | 0.002 |
| ECOM | Whooley | −0.065 | −0.139 – 0.009 | 0.085 |
| PSB | HARS | −0.191 | −0.262 – −0.119 | <0.001 |
| PSB | PCL-C | −0.258 | −0.326 – −0.187 | <0.001 |
| PSB | Whooley | −0.089 | −0.163 – −0.015 | 0.019 |
| HARS | PCL-C | 0.715 | 0.676 – 0.749 | <0.001 |
| HARS | Whooley | 0.479 | 0.419 – 0.534 | <0.001 |
| PCL-C | Whooley | 0.457 | 0.396 – 0.514 | <0.001 |

*Note: 95% confidence intervals calculated using Fisher's Z transformation. CD-RISC: Connor-Davidson Resilience Scale; ECOM: Compassion Scale; PSB: Prosocial Personality Battery; HARS: Hamilton Anxiety Rating Scale; PCL-C: Post-Traumatic Stress Checklist – Civilian version; Whooley: two-item depression screen.*
